# Consensus on draft OMERACT core domains for clinical trials of Total Joint Replacement outcome by orthopaedic surgeons: a report from the International consensus on outcome measures in TJR trials (I-COMiTT) group

**DOI:** 10.1186/s12891-017-1409-4

**Published:** 2017-01-26

**Authors:** Jasvinder A. Singh, Michael Dohm, Peter F. Choong

**Affiliations:** 1 University of Alabama at Birmingham, Birmingham, AL USA; 2 Birmingham Veterans Affairs Medical Center, Birmingham, AL USA; 3 Department of Orthopaedic Surgery, University of Arizona, Tucson, AZ USA; 4 Department of Surgery, University of Melbourne, Melbourne, Australia; 5 Department of Surgery, St. Vincent’s Hospital, Melbourne, Australia

**Keywords:** OMERACT, Core domain, Clinical trails, Total Joint Arthroplasty, TJR

## Abstract

**Background:**

There are no core outcome domain or measurement sets for Total Joint Replacement (TJR) clinical trials. Our objective was to achieve an International consensus by orthopaedic surgeons on the OMERACT core domain/area set for TJR clinical trials.

**Methods:**

We conducted surveys of two orthopaedic surgeon cohorts, which included (1) the leadership of international orthopaedic societies and surgeons (IOS; cohort 1), and (2) the members of the American Academy of Orthopaedic Surgeons’ Outcome Special Interest Group (AAOS-Outcome SIG), and/or the Outcome Research Interest Group of the Orthopaedic Research Society (ORS; cohort 2). Participants rated OMERACT-endorsed preliminary core area set for TJR clinical trials on a 1 to 9 scale, indicating 1–3 as domain of limited importance, 4–6 being important, but not critical, and 7–9 being critical.

**Results:**

Eighteen survey participants from the IOS group and 69 participants from the AAOS-Outcome SIG/ORS groups completed the survey questionnaire. The median (interquartile range [IQR]) scores were seven or higher for all six proposed preliminary core areas/domains across both groups, IOS and AAOS-Outcome SIG/ORS, respectively: pain, 8 [8, 9] and 8 [7, 9]; function, 8 [8, 8] and 8 [7, 9]; patient satisfaction, 8 [7, 9] and 8 [7, 8]; revision surgery, 7 [6, 9] and 8 [6, 8]; adverse events, 7 [5, 8] and 7 [6, 9]; and death, 7 [7, 9] and 8 [5, 9]. Respective median scores were lower for two additional optional domains: patient participation, 6.5 [5, 7] and 6 [5, 8]; and cost, 6 [5, 7] and 6 [5, 7].

**Conclusions:**

This study showed that two independent surveys dervied from three groups of orthopaedic surgeons with international representation endorsed a preliminary/draft OMERACT core domain/area set for Joint Replacement clinical trials.

## Background

Total Joint Replacement (TJR) is one of the most common elective procedures performed in the US and worldwide. The rate of utilization of this highly cost effective and clinically successful procedure has, in part, been attributed to the increasing prevalence of osteoarthritis, obesity, and an aging population [1–3]. Despite the recognition that the measurement of pain and function after TJR is very important and the common practice is to assess these outcomes in clinical practice, there is no consensus as to which domains or outcome measures should be included in every TJR trial. Consistency in measurement is essential for enabling valid comparisons between TJR clinical trials and head-to-head studies, which currently is hampered by the heterogeneity of outcome measures [4], and the inability to pool data for meta-analysis. A TJR outcome Working Group (WG) first met at the OMERACT-9 meeting in 2008 where this critical issue was discussed in detail [5]. Based on the strategy outlined, we have continued the work in this area within our WG for the last 8 years.

The method of development of TJR trial core domain set was a multi-step, data-driven process that required a consensus by key stakeholders, including but not limited to orthopaedic surgeons and patients; step-by-step details are available in a previous publication [6]. Consensus for core domains was defined as the agreement of different stakeholders (and different groups of the same stakeholder type, i.e., two groups of patients or two groups of physicians) for a similar set, if not the same, core domains. In case of a discrepancy in ratings, we would discuss with various stakeholders to agree to a final common domain set, i.e., consensus. We performed two surveys among 1) orthopaedic surgeons and 2) patients, clinicians, orthopaedic surgeons, and methodologists [7] that identified seven potential domains for a draft TJR trial core domain set (including joint-specific quality of life). At the OMERACT-2014 meeting in Budapest, a multi-stakeholder group including orthopaedic surgeons, patients, academic researchers and industry scientists, discussed in detail and by consensus endorsed a preliminary core domain set for TJR clinical trials [6]. The TJR clinical trial preliminary core domain set included six core domains, namely, pain, function, patient satisfaction, revision surgery, adverse events and death [6]. Other domains were considered but not endorsed for inclusion in the core set were patient expectations, joint-specific quality of life and generic quality of life [6]. Joint-specific quality of life was thought to be redundant with functional ability, pain and patient satisfaction and therefore not included in core domain set, details of the discussion are provided elsewhere [6]. We previously determined that the next step in this consensus process was to obtain further input from more independent groups from two important stakeholder communities, the orthopaedic surgeons and the patients.

The objective of the current study was to assess whether a broader orthopaedic community will engage in further development of the draft TJR trial core domain set, endorse or propose modification. We performed two separate surveys with three groups of orthopaedic surgeons to obtain a consensus on the draft TJR trial core domain set. We surveyed: (1) the present/past leadership of international orthopaedic societies (IOS) (survey 1); and (2) the members of the American Academy of Orthopaedic Surgeons’ Outcome Special Interest Group (AAOS-Outcome SIG) and the Outcome Research Interest Group of the Orthopaedic Research Society (ORS) at the annual meetings (survey 2), using each groups’ email list.

## Methods

### Setting

We surveyed three groups of orthopaedic surgeons, key stakeholders for this consensus, by performing two separate surveys. These surgeons included not only clinicians, but also orthopaedic clinical trialists and leading total joint registry investigators. We met orthopaedic surgeons face to face at the AAOS meeting and subsequently obtained email lists for three groups of orthopaedic surgeons, categorized as follows for two independent surveys: (1) the present/past IOS leadership (survey 1); and (2) the members of the AAOS-Outcomes SIG and the Outcome Research Interest Group of the ORS (survey 2), who attended the respective meetings at the AAOS-2015.

### Survey and analyses

We pre-tested a survey questionnaire that queried the importance of six core domains/areas (pain, function, patient satisfaction, revision surgery, adverse event and death) and two optional areas (patient participation in life/social activities, cost) for reporting in joint replacement clinical trials. Ratings were on a 1–9 scale, 1–3 indicating domain of limited importance, 4–6 being important domain, and 7–9 being critically important domain. If both groups of orthopaedic surgeons were to rate each core domain “7–9”, i.e., critical, it would indicate complete consensus, and the TJR core domain set would be considered endorsed by orthopaedic surgeons at this stage. If a majority of the six core domains, but not all, were rated as critical by both orthopaedic surgeon groups, it would indicate incomplete consensus and would lead to discussion with surgeons regarding  whether we needed modification of the draft TJR trial core domain set. In case of lack of consensus, modification of core domain set would be done. A similar approach was planned for the future, in case of lack of complete consensus between surgeon and patient surveys for the draft TJR trial core domain set, leading either to its endorsement or modification (separate surveys of the same core domain set in patients) (Singh JA, et al. Patient Endorsement of the Outcome Measures in Rheumatology (OMERACT) Total Joint Replacement (TJR) Clinical Trial Draft Core Domain Set, submitted).

Summary statistics are calculated for the two cohorts of survey participants. We calculated the median (interquartile range) ratings for each of the preliminary core domain/area within each group, the orthopaedic surgeon/registry leadership group and the AAOS-Outcome SIG/ORS orthopaedic surgeon groups.

## Results

There were 18 survey participants from the IOS group and 69 participants from the AAOS-Outcomes SIG/ORS outcome groups who completed the questionnaire. Of the survey respondents, respectively, 100 and 78% were male, >90% in both were arthroplasty researcher/surgeons, and 67 and 64% were 55 years and older (Table 1).

**Table 1:** Participant characteristics

# survey participants	Orthopaedic surgeon leadership group	Orthopaedic surgeons (AAOS outcomes SIG/ORS group)
	*N* = 18	*N* = 69
% male^a^	100%	78%
Age category^a^		
18–24	0%	0%
25–34	0%	1%
35–44	0%	17%
45–54	33%	17%
55–64	67%	41%
65–74	0%	19%
≥75	0%	4%
Background^b^		
Arthroplasty surgeon, private practice	56%	18%
Arthroplasty surgeon, academic practice	39%	43%
Arthroplasty researcher	11%	49%
Orthopaedic surgeon, not focused on arthroplasty, private practice	11%	8%
Orthopaedic surgeon, not focused on arthroplasty, academic practice	17%	7%
Patient	0%	7%
Policy maker	0%	3%
Time spent planning/conducting arthroplasty trials		
0–10%	67%	57%
11–20%	28%	16%
21–30%	0%	16%
31–50%	0%	4%
>50%	6%	6%
Years practicing/doing research in arthroplasty		
< 5	6%	4%
5–<10	0%	13%
10–20	18%	23%
>20	77%	59%

^a^Missing values for AAOS-Outcome SIG and OMERACT cohorts: sex: 5 vs. none; age, 4 vs. none; ^b^Background, people could choose more than one option

The pattern of endorsement and ratings of both core domains and optional domains for measurement in arthroplasty RCTs were similar across the two diverse groups of orthopaedic surgeons (Table 2). All six proposed preliminary core domains of pain, function, patient satisfaction, revision surgery, adverse event and death achieved a median score of 7 or 8 on a 0–9 scale in both groups, i.e., were rated as critically important. The median score for two additional optional domains, patient participation and cost, achieved a median score of 6–6.5 in both groups (Table 2).

**Table 2:** Preliminary core areas/domains for TJR clinical trials

	Orthopaedic surgeon leadership group Median [IQR]	Orthopaedic surgeons (AAOS, ORS group) Median [IQR]
Main Domains to be reported in every TJR clinical trial		
Joint Pain	8 [8, 9]	8 [7, 9]
Function or functional ability (ability to function in society, work; work productivity, employability; disability; work disability)	8 [8, 8]	8 [7, 9]
Patient Satisfaction (satisfaction with the outcome, satisfaction with the procedure)	8 [7, 9]	8 [7, 8]
Revision surgery	7 [6, 9]	8 [6, 8]
Adverse events	7 [5, 8]	7 [6, 9]
Death	7 [7, 9]	8 [5, 9]
Additional domains for consideration for reporting in TJR clinical trials		
Patient Participation	6.5 [5, 7]	6 [5, 8]
Cost	6 [5, 7]	6 [5, 7]

Several important comments were provided by survey respondents, which shed further insight into this issue (Table 3). Additional core domains brought up for consideration by 1 respondent each were: caregiver impact; venous thromboembolism occurrence; return to work; quality of life; and range of motion. Some participants commented that the domains of revision (at some specified time/s after TJR) and patient participation (social vs. work) need to be defined clearly.

**Table 3:** Additional Comments from Delphi Process

Main core domains	Additional domains
AAOS-Outcomes SIG/ORS Orthopaedic surgeon group	
Change in function, change in pain duration of improvement	Cost is a very nebulous area, particularly since cost varies by how you measure it and what part of the country the research is performed in
Patient satisfaction is nebulous unless everyone is using the same instrument to measure it.	I am not sure what patient participation means, so I stayed neutral. I suspect this refers to charges and in that case, there needs to be a cost to charge ratio applied
I believe that limping is important and has a tendency to drown in the available scores used	Limping for total hip and stiffness (joint fibrosis) for TKA
Patient-reported outcomes using validated instruments should have a very high priority and would cover the upper (first) two items.	Unclear what is meant by patient participation so unable to score
PROM function and patient satisfaction are important but both are often affected by factors not directly related to the outcome of surgery. Thus it depends on the type of clinical trial if they are essential or not. Revision surgery indicates that both the patient and the physician agreed on that the initial procedure had been unsuccessful, Death and adverse events are very important but uncommon. For a new technique or approach such information is essential but maybe not for every clinical trial.	Participation in rehabilitation is important to functional outcome, but the trial focus may or may not require collection of these data. If patients are randomized to a drug trial, for example, then participation levels should be random and the focus is on the endpoints/outcomes (pain, function, AE) not on the level of participation.
Family function; Caregiver impact	Medical and surgical complications should be reported separately
Venous thromboembolism occurrence–What is the Best VTE prophylaxis?	Need more definition around how cost will be measured and reported Cost is variable from state to state and center to center within a state or city
Revision should probably be re-designated as “reoperation for any reason” as patients often care more if they had to have repeat surgery for what ever the reason, and not as much whether implant parts (if any) are swapped out or exchanged	Patient participation is very unreliable on higher levels of participation
Implant revision is impractical unless the trial involves many years of follow-up. However, surgical AEs (manipulation, etc.) are important. Death is important, but very rare	
Of importance for the patient: To which degree are expectations fulfilled	
Return to work or avoidance of environment modification for the older patient	
Quality of life, 8 Work disability and employability are very culture- and society-dependent issues and therefore suit poorly as core domains. Function should be assessed in more general and patient-specific terms	
Range of motion	
Patient Satisfaction should not be a core domain and should not be reported by all arthroplasty trials should be optional	
Longevity of arthroplasty–similar to revision surgery	
For the first two listed (joint pain and function), I think it would be very important to measure those outcomes not only at 6–12 months after surgery, but also at an earlier time interval after surgery (ex. 6 weeks) for patients interested in the extent to which they can expect rapid recovery.	
My rating is in part weighted by how accessible the information is. For example, revision surgery or death may take place years or decades after the initial surgery, and hence may not be related or the index procedure may not be available to the researcher. Secondly, there is little consensus of what is a “revision surgery”. A German colleague demonstrated that an I & D was considered a revision surgery in France, but not in other countries. Similar for “adverse events”. Would this include a post-op infection resulting in an infection? Obviously you have a lot of work here—what periods are being suggested? I know that the spine surgeons may consider 2 years as acceptable follow-up. Not sure what this group is considering. 1 year? 2 years? 5 years? At what point is the patient’s status no longer relevant to the surgery? Good luck Mike. These are my perspectives and may not be representative of the physicians involved. Stan	
Orthopaedic leadership group	
The importance of death is social situation dependent. For two identical patients but for one having dependent family and the other not, the impact of death would be different, and might affect the decision to have surgery	Cost should be cost-effectiveness. These two terms are not necessarily interchangeable and there is an important difference in arthroplasty. A cheaper cemented stem and cup in a young person is not cost effective
Death as long as related to surgery. Patient satisfaction probably one question item. Link revision to joint registries if they exist in the country–a better question may be any other surgery related to joint where revision surgery not an option (i.e. ankle arthroplasty)	Cost is such a veritable worldwide. It will be difficult to quantify it accurately based on the various deals and accountancy practices in different hospitals. I’m not sure as part of a clinical review of the performance
I believe patient satisfaction and function of secondary only to the relief of osteoarthritic pain.. Adverse events I think a slightly less important as they reflect on the anesthetic care and this is going ability rather than the prosthetic	
Function domain needs to be relative to the involved joint and attempt to capture non joint limiting disabilities	

## Discussion

TJR clinical trials often report outcomes that are heterogeneous and difficult to combine and compare across trials in a systematic review or comparative analysis [4, 5]. This heterogeneity is not only a major barrier to accurate comparison between trials but also an impediment to the development of consensus, which is critical for the collective analysis of the important findings that TJR trials may bring. The various international joint replacement registries have recognized the importance of harmonizing outcome collection and reporting based on expert consensus and have initiated a collaborative approach to establishing a framework [8]. To date, such an effort has not been completed for a core measurement set in TJR clinical trials.

The OMERACT TJR Working Group, an International group with patient partnership, is pursuing this goal through International collaboration with groups such as the AAOS-Outcome SIG, the ORS, the International Society of Arthroplasty Registries (ISAR), and Functional Outcomes in Total Joint Replacement (FORCE-TJR). In the last few meetings, the OMERACT TJR WG has made significant progress in assembling a multi-stakeholder group and completed the literature reviews examining the frequency of reporting of various domains (pain, function, revision etc.) in TJR clinical trials (Richards B, et al. Outcome measures used in arthroplasty trials: Systematic review of the 2008 and 2013 literature, submitted; Wall P, et al. Do outcomes reported in randomised controlled trials of joint replacement surgery fulfil the OMERACT 2.0 Filter? A review of the 2008 and 2013 literature, submitted).

At OMERACT 2014 TJR WG, a preliminary core domain set for TJR clinical trials was developed based on systematic literature reviews, the OMERACT filter 2.0, and two separate surveys at orthopaedic surgery meetings and the OMERACT pre-meeting [6]. This proposed draft TJR core domain set (pain, function, patient satisfaction, revision, adverse events, death, joint-specific quality of life) was discussed with a multi-stakeholder group at OMERACT-2014 TJR WG that included patients who had undergone arthroplasty, orthopaedic surgeons (in an academic setting or community-based practice), psychometricians, physical/occupational therapists, other clinicians and arthroplasty researchers, and finally endorsed with six core domains [6]. This multi-stakeholder OMERACT group helped develop the draft TJR clinical trial core domain set including experts in the development of clinical trial core domain sets and following a well-described methodology based on a solid framework of the OMERACT filer 2.0 [9–11]. 

In the current study, both groups of orthopaedic surgeons achieved consensus on the six TJR clinical trial core domains (scores of 7 or above) and did not propose any new core domains. These are very important findings, since these Delphi involved two groups of orthopaedic surgeons independent of those who had participated in the development of the preliminary TJR clinical trial core domain set. This is not surprising since this TJR core domain set was developed over several years after multiple systematic reviews and with multi-stakeholder input consisting of orthopaedic surgeons, patients who had undergone arthroplasty and methodologists who have an extensive experience with developing and validating core domain and measurement sets. The OMERACT’s philosophy of a multi-stakeholder involvement from the beginning of the process, and a data driven approach, are key to successfully achieving a broad stakeholder consensus on this draft TJR clinical trial core domain set. This evidence indicates that preliminary TJR core domain set initially developed with multi-stakeholder input and endorsed at OMERACT 2014 meeting was endorsed independently by orthopaedic surgeons.

Core domain sets are those domains that should be  reported in every clinical trial for the condition, regardless of the intervention, as they are critical to the conceptualization of the condition/disease. One must not confuse a core domain with the primary outcome for each clinical trial, which is always specific to each research question. In some studies, core domain measures will be primary outcome, since they are the focus of the research question; in other studies, they may be secondary study outcomes. Our work indicates that this draft TJR core domain set is now ready for the next phase, namely, the identification and validation of measures that accurately reflect each core domain, and further dissemination of this TJR core domain set to key stakeholders.

We received qualitative feedback from the surveys, which has helped us with further specification of two core domains. One suggestion was to consider any reoperation, not just revision, due to its relevance to patients- this indicates that the core domain would be revision/reoperation. Another suggestion was to specify whether death was procedure-related vs. not procedure-related. With further refinement of the core domains, these two proposals will be the subject of future WG discussions. It is interesting and reassuring that several of domains brought up one participant each (caregiver impact; venous thromboembolism occurrence; return to work; quality of life; and range of motion) were considered previously. Based on Delphi and detailed discussions with surgeons, patients and other stakeholders, they were recommended not for inclusion in the core domain set, since they were not considered to represent an independent core domain, above and beyond the six domains already included.

We noted that both groups independently rated the additional non-core domains (patient participation, cost) as important but not critical; the median scores were 6 to 6.5, appropriately lower than the core domains, since these are not core domains. Non-core domains are those domains that may be additionally reported in a TJR clinical trial, depending on the clinical question being asked.

Our study findings must be interpreted with caution, since they may not be generalizable to other groups. Our orthopaedic surgeon samples were 78 and 100% male, which may limit the generalizability of the findings to all surgeons. The gender distribution among orthopaedic surgeons is not balanced in general. We developed this draft TJR core domain set for clinical trials; it is possible that with validation and future work, this set or a similar set may be endorsed for observational studies as well.

Our study has several strengths, including use of the same survey in two groups of stakeholders, inclusion of both leaders of orthopaedic societies as well as the group of orthopaedic surgeons and researchers from community and academic practices.

## Conclusions

In conclusion, the current study shows further independent endorsement of the draft TJR core domain set for reporting of TJR clinical trials. The next steps in this process, are a wider endorsement of these by OMERACT and/or orthopaedic organizations that we have been collaborating with. This will be  followed by the development of a valid, data- and consensus-driven TJR clinical trial core outcome measurement set. Our group will aim to complete this step over the next 2 years. The process will be completed with multi-stakeholder input with a particular emphasis of involvement of patients and TJR surgeons.

## Abbreviations

AAOS: American Academy of Orthopaedic Surgeons’ Outcome Special Interest Group
NRS: Numeric Rating Scale
OA: Osteoarthritis
OMERACT: Outcome measures in rheumatology
ORS: Outcome Research Interest Group of the Orthopaedic Research Society
SIG: Special Interest Group
TJR: Total Joint Replacement
TKA: Total knee arthroplasty
WG: Working group
WOMAC: Western Ontario and McMaster Universities Osteoarthritis Index
